# Chemometric Analysis of Low-field ^1^H NMR Spectra for Unveiling Adulteration of Slimming Dietary Supplements by Pharmaceutical Compounds

**DOI:** 10.3390/molecules25051193

**Published:** 2020-03-06

**Authors:** Nao Wu, Stéphane Balayssac, Saïda Danoun, Myriam Malet-Martino, Véronique Gilard

**Affiliations:** Groupe de RMN Biomédicale, Laboratoire SPCMIB (UMR CNRS 5068), Université Paul Sabatier, Université de Toulouse, 118 route de Narbonne, 31062 Toulouse Cedex, France; nao.wu@chimie.ups-tlse.fr (N.W.); danoun@chimie.ups-tlse.fr (S.D.); martino@chimie.ups-tlse.fr (M.M.-M.)

**Keywords:** dietary supplement, adulteration, low-field NMR, multivariate analysis

## Abstract

The recent introduction of compact or low-field (LF) NMR spectrometers that use permanent magnets, giving rise to proton (^1^H) NMR frequencies between 40 and 80 MHz, have opened up new areas of application. The two main limitations of the technique are its insensitivity and poor spectral resolution. However, this study demonstrates that the chemometric treatment of LF ^1^H NMR spectral data is suitable for unveiling medicines as adulterants of slimming dietary supplements (DS). To this aim, 66 DS were analyzed with LF ^1^H NMR after quick and easy sample preparation. A first PLS-DA model built with the LF ^1^H NMR spectra from forty DS belonging to two classes of weight-loss DS (non-adulterated, and sibutramine or phenolphthalein-adulterated) led to the classification of 13 newly purchased test samples as natural, adulterated or borderline. This classification was further refined when the model was made from the same 40 DS now considered as representing three classes of DS (non-adulterated, sibutramine-adulterated, and phenolphthalein-adulterated). The adulterant (sibutramine or phenolphthalein) was correctly predicted as confirmed by the examination of the ^1^H NMR spectra. A limitation of the chemometric approach is discussed with the example of two atypical weight-loss DS containing fluoxetine or raspberry ketone.

## 1. Introduction

Nowadays, the adulteration of dietary supplements (DS) with approved or non-approved medicines poses a threat to the health of consumers [1]. The adulteration of slimming preparations with active pharmaceutical ingredients to increase their effects is a widely reported issue [2]. The two most common adulterants detected in weight-loss preparations are sibutramine and phenolphthalein, alone or in combination [3,4,5]. Sibutramine is an anorectic drug that was withdrawn from the market of many countries (European Union, USA, China, Australia, India …) since 2010 because of cardiovascular concerns. Phenolphthalein is used for its laxative properties even though it has been removed from over-the-counter products in the late 1990s due to a carcinogenic risk [6].

Various analytical methods have been proposed for the detection and/or quantification of undeclared drugs in slimming DS. The most frequently described technique is liquid chromatography with ultraviolet, diode-array or mass spectrometry detection [7,8,9]. Other methods including vibrational spectroscopy [10], gas chromatography [4] or ion exchange chromatography with conductivity detection have been proposed [11]. High-Field (HF) ^1^H NMR spectroscopy has also been successfully applied for detecting and quantifying adulterants in slimming DS [5].

Low-field (LF) NMR is an emerging technique based on the use of a new generation of compact NMR [12,13,14]. A few applications of LF NMR in the pharmaceutical field have recently been described [15,16,17,18,19] and the feasibility of LF NMR for unveiling adulteration of DS has been demonstrated [15,19].

The aim of the present study is to deepen the evaluation of LF NMR to detect adulteration of slimming DS by coupling LF ^1^H NMR data with a chemometric analysis, thus allowing classification of samples without expert interpretation of NMR spectra recorded on a low-cost benchtop spectrometer. We have thus analyzed adulterated and non-adulterated slimming DS, previously qualitatively and quantitatively characterized by HF ^1^H NMR [5], with LF ^1^H NMR in order to create statistical models in which the LF ^1^H NMR data of new samples are injected. The interest and limitations of this approach are discussed.

## 2. Results and Discussion

### 2.1. LF ^1^H NMR Analysis

The weight-loss DS used in this study, except the newly purchased test samples (T), were previously analyzed and fully characterized by HF ^1^H NMR, i.e., the nature and amount of adulterants by unit (capsule, tablet, or sachet) were known [5]. The full list of DS is given in Table S1.

**Figure 1:**
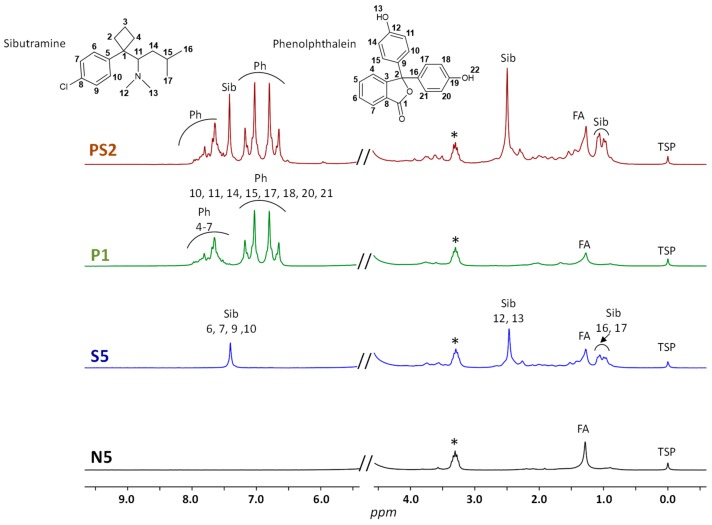
Typical LF ^1^H NMR spectra of weight-loss dietary supplements recorded at 60 MHz (N, non-adulterated (natural) group; S, sibutramine-adulterated group; P, phenolphthalein-adulterated group; PS, both sibutramine and phenolphthalein-adulterated group). Ph: Phenolphthalein; Sib: Sibutramine; FA: Fatty acids; TSP: Internal reference; *: CD_2_HOD.

In the first step of the present study, all DS were analyzed in duplicate by LF ^1^H NMR in deuterated methanol. The recording time of each spectrum was 15.5 min, and the profiles of typical samples are illustrated in Figure 1.

Although LF ^1^H NMR spectra are rather poorly resolved, the main characteristic signals of sibutramine and phenolphthalein, the two most common adulterants of slimming DS, are easily detected alone or in combination. As it can be seen in Figure 1, sibutramine is identified in samples S5 and PS2 by the signals of its aromatic protons at 7.41 ppm and of its methyl groups at 2.49 (CH_3_ 12 and 13) and 1.02 (CH_3_ 16 and 17) ppm. Likewise, aromatic protons of phenolphthalein give a characteristic pattern (6.5-8.0 ppm) that can be observed in DS P1 and PS2. Sample N5 is a DS without adulterant and, except for the reference and solvent signals, only the signal of some CH_2_ protons of fatty acids from plant extracts is readily detected at 1.27 ppm. Minor signals corresponding to aromatic protons of natural polyphenols or other natural compounds are also detected in a few samples.

### 2.2. Chemometric Analysis

To start the chemometric analysis, a statistical model was built by performing a two-class comparison: DS without adulterant (natural: N, *n* = 19) were compared to DS containing either sibutramine (S, *n* = 12) or phenolphthalein (P, *n* = 9), samples (S) and (P) being considered together (*n* = 21) as “adulterated samples”. After spectra processing (6–8 ppm region, see experimental part), bucketing and normalization of the data, the Partial Least Squares-Discriminant Analysis (PLS-DA) led to a predictive model with two principal PLS components and good validation criteria (*Q*^2^ = 0.61, *R*^2^*Y* = 0.76, *CV-ANOVA* = 2.3 × 10^−18^). All Q^2^ and R^2^ values were lower in the permutation test than in the model, confirming its goodness. The classification of all samples was then obtained from the two-class model based on the predicted Y-values (YpredPS, which is the Y value predicted by the model based upon the X block variables (resonance intensities at given ppm)) indicating the probability that a sample belongs to one class of the model (adulterated or non-adulterated).

**Figure 2:**
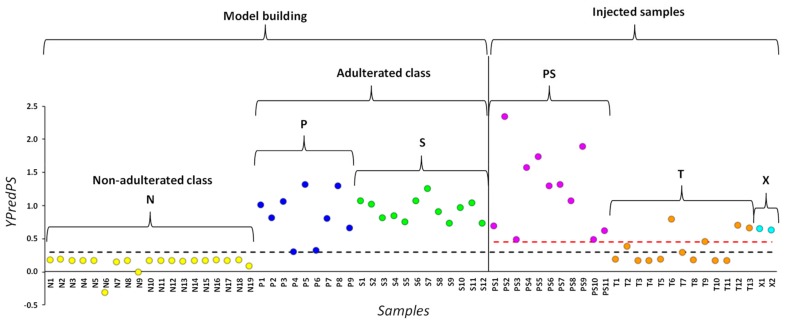
Predicted Y-values (YpredPS) obtained for the 66 DS analyzed based on the two-class PLS-DA model comparing natural samples (N) to adulterated samples (samples (P) and (S) considered together as a single class of adulterated samples). Samples above the red dashed line (*YpredPS* = 0.45) are defined as adulterated and those below the black dashed line (*YpredPS* = 0.30) as natural. PS, both sibutramine and phenolphthalein-adulterated group; T: test samples, i.e., newly purchased DS; X: atypical DS.

YpredPS values for the 66 DS analyzed in this study are reported in Figure 2. Samples (N), (S) and (P) (*n* = 40), whose content was previously known [5], were considered for the definition of a Y-value threshold between adulterated and non-adulterated DS. An YPredPS value close or superior to 1 would indicate that the sample is likely to belong to the adulterated class while an YPredPS value close to 0 would indicate that the sample is likely to be natural. Conventionally, a threshold of 0.65 was defined for samples belonging to a defined class and a 0.65–0.35 range for samples borderline to the defined class [20]. In our study, it appears that the adulterated samples (P) and (S) have YpredPS > 0.65, except P4 and P6 whose YpredPS are 0.30 and 0.32 respectively, whereas the highest YpredPS for (N) samples is 0.18. Based on the knowledge of the content of these two DS, we thus defined the lowest limit of the threshold at 0.30 (black dashed line in Figure 2). Samples (PS) previously shown as adulterated by both sibutramine and phenolphthalein were then injected into the model. Their YpredPS values being > 0.47, the upper limit of the threshold was set at 0.45 (red dashed line in Figure 2). So, DS with YpredPS values > 0.45 were considered as belonging to the adulterated class, those with YpredPS values < 0.30 to the non-adulterated class, and samples with YpredPS values between 0.30 and 0.45 were considered as borderline.

If we apply these criteria to the newly purchased DS (T, test samples), the classification shows that samples T6, T9, T12, and T13 are predicted adulterated, and samples T2 and T7 borderline, whereas other T samples are predicted natural with Y values ≤ 0.18 (Table 1). Two atypical DS, X1 (*YPredPS* = 0.65) and X2 (*YPredPS* = 0.63), which will be discussed later, are predicted adulterated by the two-class model. In conclusion, this preliminary rapid analysis with the two-class PLS-DA model can be considered as a first screening of adulterated slimming DS leading to a classification between natural, adulterated or possibly adulterated (borderline) samples.

**Figure 3:**
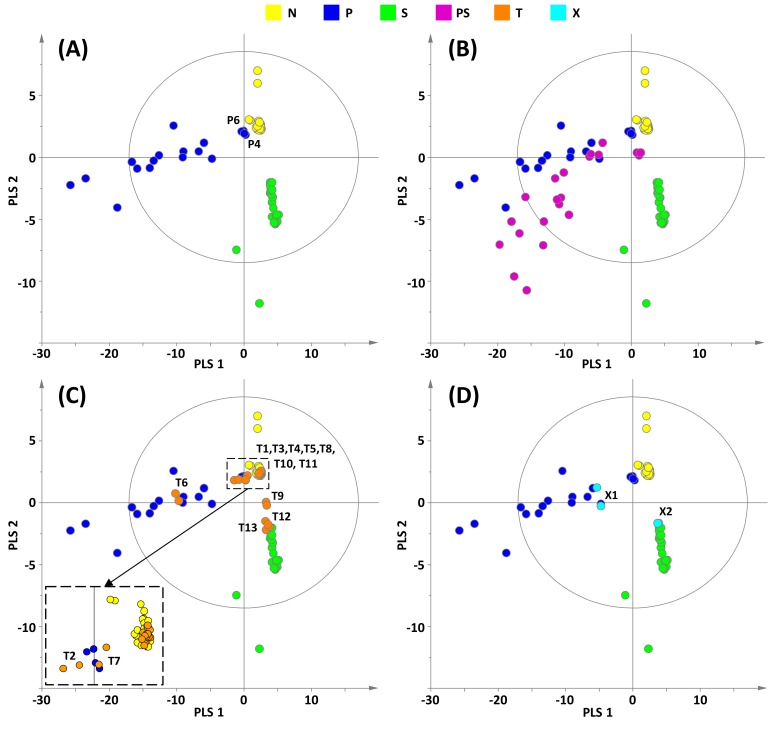
(**A**) Score plot of the PLS-DA three-class model built from LF ^1^H NMR spectra of samples N (non-adulterated), S (adulterated with sibutramine), and P (adulterated with phenolphthalein). Score plots (**B**), (**C**) and (**D**) show the projection of samples PS (adulterated with both sibutramine and phenolphthalein), T (test samples) and X (atypical samples, see text) respectively on the built model (A).

To go further in the classification of the DS, a new PLS-DA analysis was carried out in which samples (N), (P) and (S) were considered as three distinct groups. A good predictive model was obtained with two principal PLS components (*Q*^2^ = 0.66, *R*^2^*Y* = 0.74), a p-value of the CV-ANOVA of 3.4 × 10^−21^, and a permutation test successfully performed. The score plot of this three-class PLS-DA shows a clear discrimination between the three categories of DS (Figure 3A). Samples (P) (dark blue) and (S) (green) appear more spread out than samples (N) (yellow) because of the variable amount of adulterant in each sample ranging from 8 to 16 mg per unit for sibutramine in samples (S) and from 5 to 55 mg per unit for phenolphthalein in samples (P) [5].

(PS) samples (purple) projected in this three-class PLS-DA model are located closer to (P) than to (S) samples (Figure 3B). This observation is in agreement with higher amounts of phenolphthalein compared to sibutramine contained in most samples [5]. The score plot of the projection of test samples (T) in the model confirms the classification proposed in Table 1 but affords a more precise analysis (Figure 3C). Indeed, samples T1, T3–5, T8, T10, and T11 overlap with (N) samples and can thus be considered as natural. Samples T9, T12, and T13 contain the adulterant sibutramine whereas samples T2, T6, and T7 contain phenolphthalein. It can be noticed that none of the (T) samples belong to the (PS) class, i.e., contain a mixture of phenolphthalein and sibutramine. As the statistical analysis of the (T) samples was done blindly, i.e., without a thorough examination of their LF (and HF) ^1^H NMR spectra, these findings were confirmed by the visual analysis of these spectra.

**Figure 4:**
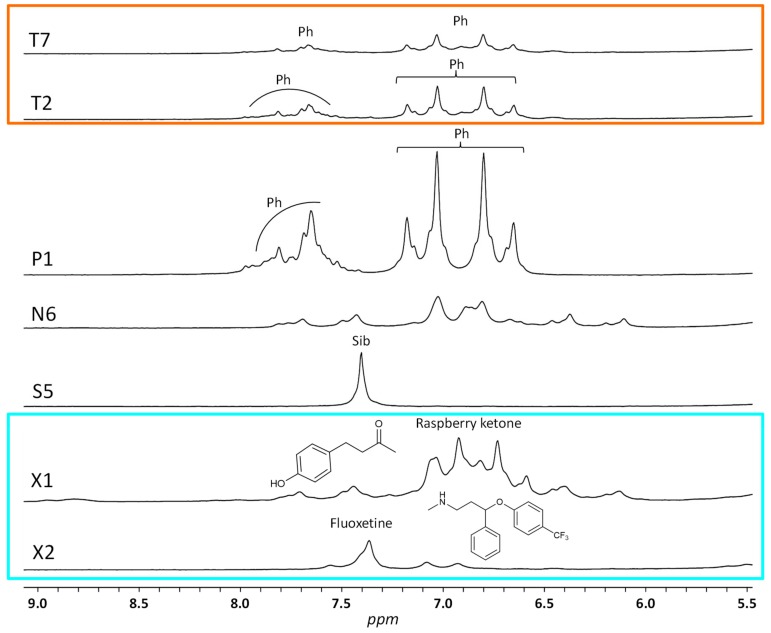
LF ^1^H NMR spectra of some weight-loss dietary supplements recorded at 60 MHz. Ph: phenolphthalein; Sib: sibutramine.

The fact that the two samples T2 and T7 were considered as borderline in the classification established from the predicted Y-values of the previous two-class model (Table 1) but are now better characterized by the three-class model (Figure 3C) can also be explained by the visual observation of their LF ^1^H NMR spectra. Indeed, as reported in Figure 4, signals of phenolphthalein are detected in samples T2 and T7 but with a lower signal-to-noise ratio than in the P1 spectrum due to the low amount of adulterant in these DS. We mentioned above that signals corresponding to aromatic protons of natural polyphenols or other natural compounds were detected in a few (N) samples (as an illustration, the LF ^1^H NMR spectrum of sample N6 is shown in Figure 4). Their chemical shifts and intensities close to those observed for phenolphthalein in T2 and T7 samples led to the classification of these DS as borderline in the first approach (Table 1).

A limitation of the present work is illustrated by the examples of the two DS X1 and X2. These samples appear as adulterated when considering their predicted Y-values (0.65 for X1 and 0.63 for X2) (Figure 2). Moreover, the projection of their LF ^1^H NMR spectra in the PLS-DA three-class model indicates that the adulterants are phenolphthalein for X1 and sibutramine for X2 (Figure 3D). In fact, we demonstrated in a previous HF ^1^H NMR study [5] that these two samples contain respectively raspberry ketone, a natural phenolic compound (probably intentionally added due to its high concentration in this particular DS), and fluoxetine, an antidepressant drug illegally added. As the model was built with only the LF ^1^H NMR data of DS (N), (S) and (P), the chemometric analysis leads to the misclassification of X1 and X2. The reason can be found in their LF ^1^H NMR spectra (Figure 4). Indeed, the main aromatic signal of fluoxetine has a chemical shift (7.37 ppm) close to that of sibutramine (7.41 ppm) and the large aromatic multiplet of raspberry ketone overlaps with the resonances of phenolphthalein. Although chemical shifts of these chemicals are slightly different from those of sibutramine or phenolphthalein, the multi-alignment procedure applied prior to the statistical treatment results in their misclassification. Nevertheless, even if the adulterant statistically identified in X2 is not the good one (i.e., sibutramine instead of fluoxetine), this DS remains unsafe for the consumer and the goal of the statistical screening for detecting dubious samples is thus achieved.

In conclusion, the three-class PLS-DA works well as it enables a correct prediction of the nature of the adulterant sibutramine or phenolphthalein, the two banned drugs most commonly added to weight-loss DS to improve their effectiveness. The lowest limit of phenolphthalein concentration detected by the model is 3 mg per 100 mg of powder, which corresponds to ≈ 6 mg per unit if a mean capsule content weight of 200 mg is considered. The lowest limit of sibutramine concentration could not be reached because all the analyzed DS had YPredPS values > 0.7, very far from the 0.3 value that characterizes the limit between adulterated and non-adulterated DS (Figure 2). A source of classification error is nevertheless possible if an adulterant or a natural compound has a structure leading to ^1^H NMR signals in the resonance frequency areas considered to build the model. For example, the characteristic signal of the methyl protons 16 and 17 of sibutramine at 1.02 ppm could not be used to create the model because it was often overlapped with the resonance of fatty acids.

This study shows that applying a chemometric treatment to LF ^1^H NMR data is a means of widening the field of application of the technique, in particular for the analysis of complex mixtures. This approach has been successfully proposed in agri-food applications for the analysis and authentication of edible oils [21,22] and meat [23]. Very recently, a similar approach was used for the analysis of substandard and falsified medicines [19]. Our study expands the field to the adulteration of DS, a problem at the crossroads between agri-food and health products. In the case of slimming DS adulteration, the analytical process proposed can be useful for the first-line detection of samples liable to be adulterated without resorting to expert analysis of the ^1^H NMR spectra. Sample preparation is simple and fast, and LF ^1^H NMR acquisition is easy, quite push-button and does not require specific NMR knowledge. The perspective of this study would be to automate the whole process in order to propose a turnkey method that could be implemented in quality control labs.

## 3. Materials and Methods

### 3.1. Samples

Different groups of weight-loss DS were ordered on the Internet and analyzed with LF ^1^H NMR: (N) without adulteration, (S) adulteration with sibutramine, (P) adulteration with phenolphthalein, (PS) adulteration with both phenolphthalein and sibutramine, (T) test samples, and (X) two atypical samples (Table S1). The 40 DS used for building the statistical models ((N) (*n* = 19), (S) (*n* = 12), and (P) (*n* = 9)) as well as (PS) samples (*n* = 11), and the two DS (X) were previously qualitatively and quantitatively characterized in our lab with HF ^1^H NMR [5]. For testing the statistical models, 13 new DS (T1–T13) were bought on the Internet in November 2019 and were analyzed by LF and HF ^1^H NMR upon receipt.

### 3.2. Sample Preparation for LF ^1^H NMR Analysis

Around 100 mg of powdered samples were mixed with 1 mL of deuterated methanol under vortex agitation for 15 s and then sonicated for 5 min. The suspension was then centrifuged (5 min, 3000 rpm) and the supernatant (700 µL) analyzed. Thirty microliters of sodium 2,2,3,3-tetradeutero-3-(trimethylsilyl) propanoate (TSP, 40 mM) as the internal chemical shift reference was added before the NMR analysis. Each DS was prepared in duplicate.

### 3.3. LF ^1^H NMR Analysis

Qualitative LF ^1^H NMR spectra were acquired on a Pulsar™ benchtop NMR spectrometer (Oxford Instruments, Abingdon, UK) operating at a frequency of 59.7 MHz for ^1^H. The temperature inside the spectrometer was 310 K. The acquisition was performed by using the SpinFlow 1.2.0.1 software (Oxford Instruments) and the processing was done with MNova 11.0 (Mestrelab Research, Santiago de Compostela, Spain). Free induction decays (FIDs) were recorded with a flip angle of 90° (12.5 μs), a spectral width of 5000 Hz (83.75 ppm), and 8 K complex points (acquisition time of 1.64 s). The relaxation delay was set at 2 s, and 256 transients were recorded leading to a total acquisition time of 15.5 min. For data processing, the FIDs were apodized with an exponential filter (line broadening (LB) of 0.3 Hz), and a Whittaker smoother was applied for automatic baseline correction. The number of points was increased to 16 K in Fourier transformed spectra. The signal of TSP set at 0 ppm was used as an internal reference for chemical shift (δ) measurement.

### 3.4. Chemometrics

First, the data matrix with all LF ^1^H NMR spectra (132 spectra) of ((N), (S), (P), (PS), (T), and (X)) groups was generated in the Chemometrics module included in the MNova software with a spectral resolution of 0.01 ppm/point. Data were transferred to the Matlab^®^ software (R2018a, The Mathworks Inc., Natick, MA, USA) for the alignment procedure using the Icoshift algorithm [24] with the following input arguments: PS9 as reference spectrum (target vector), data matrix with all spectra, a file for local alignment with three specific intervals 8.385–7.495, 7.495–7.275, and 7.275–6.055 ppm, an optional ‘f’ command for a fast search of the best alignment for each interval and no co-shift preprocessing step. Then, the bucketing procedure was performed with an optimized bucketing algorithm [25] and a fixed bin width of 0.01 ppm. Data were normalized by dividing their areas by that of the internal standard TSP signal and by the weight of powder in capsules, tablets, sachet, coffee or tea bags. Multivariate statistical analyses were done with the SIMCA-P+ 13.0 software (Umetrics, Umea, Sweden). PLS-DA with UV-scaling analyses were performed with two (80 spectra corresponding to 19 samples (N) and 21 adulterated samples (P) and (S)) or three qualitative variables (19 (N), 9 (P) and 12 (S)). Then (PS), (T) and (X) LF ^1^H NMR data (52 spectra) were projected into the active model and predicted score plots were built. Predicted Y-values (YPredPS) were provided by the classification list included in the predict module of the SIMCA-P+ software.

## Figures and Tables

**Table 1:** Classification list showing predicted Y-values (YPredPS) for test samples (T) based on the two-class PLS-DA model built with LF ^1^H NMR data and completed by the visual observation of the projection of the samples on the three-class PLS-DA model shown in Figure 3A.

Identification	Predictive Y-value Classification from the Two-Class PLS-DA		Projection on the Three-Class PLS-DA Model Shown in Figure 3A	
	YPredPS	Classification	Class membership	Adulterant
T1	0.18	natural	N	-
T2	0.37	borderline	P	phenolphthalein
T3	0.16	natural	N	-
T4	0.17	natural	N	-
T5	0.18	natural	N	-
T6	0.79	adulterated	P	phenolphthalein
T7	0.30	borderline	P	phenolphthalein
T8	0.17	natural	N	-
T9	0.45	adulterated	S	sibutramine
T10	0.17	natural	N	-
T11	0.17	natural	N	-
T12	0.69	adulterated	S	sibutramine
T13	0.65	adulterated	S	sibutramine

## Supplementary Materials

The following are available online at https://www.mdpi.com/1420-3049/25/5/1193/s1. Table S1. Information on slimming dietary supplements analyzed in this study.
