# Supplementary material for: Chemometric Analysis of Low-field 1H NMR Spectra for Unveiling Adulteration of Slimming Dietary Supplements by Pharmaceutical Compounds
Source: Molecules. 2020 Mar 6;25(5):1193. doi: 10.3390/molecules25051193 (PMC7179456; doi:10.3390/molecules25051193)
Supplement: Supplementary file 1 [file molecules-25-01193-s001.pdf]

Supplementary Material

Chemometric Analysis of Low-field ^{1}H NMR Spectra for Unveiling Adulteration of Slimming Dietary Supplements by Pharmaceutical Compounds

Nao Wu, Stéphane Balayssac *, Saïda Danoun, Myriam Malet-Martino and Véronique Gilard *

Groupe de RMN Biomédicale, Laboratoire SPCMIB (UMR CNRS 5068), Université Paul Sabatier, Université de Toulouse, 118 route de Narbonne, 31062 Toulouse cedex, France; nao.wu@chimie.ups-tlse.fr (N.W.); danoun@chimie.ups-tlse.fr (S.D.); martino@chimie.ups-tlse.fr (M.M.-M.)

* Correspondence: balayssac@chimie.ups-tlse.fr (S.B.); gilard@chimie.ups-tlse.fr (V.G.); Tel.: +33-5-61-55-82-81

Table S1. Information on slimming dietary supplements analyzed in this study.

Identification[1]	Formulation name	Form	Adulterant	
			Sibutramine	Phenolphthalein
N1	7+1	Capsule		
N2	AF6	Capsule		
N3	CiblAction	Capsule		
N4	Colodetox	Capsule		
N5	KILO KO	Capsule		
N6	Kilo-Minus	Capsule		
N7	Slim effect 24h	Powder sachet		
N8	Slim fast	Capsule		
N9	Slimula	Capsule		
N10	Cacti-Nea	Tablet		
N11	Fruta bio	Capsule		
N12	Instant slim	Capsule		
N13	La Jiao Shou Shen	Capsule		
N14	Lipo Bomb	Capsule		
N15	Natural Max Slimming	Capsule		
N16	PAI YOU GUO	Capsule		
N17	Pure fat 3 days	Capsule		
N18	Sleep and slim	Capsule		
N19	Slimming essence	Capsule		
P1	1 day diet	Capsule		×
P2	Majestic Slimming capsule	Capsule		×
P3	Royal Slim	Capsule		×
P4	Citrus'Fit	Capsule		×
P5	Xiushentang	Capsule		×
P6	Xiyoujiqingzhi capsule	Capsule		×
P7	Body beauty	Coffee bag		×
P8	Green coffee 1000	Coffee bag		×
P9	Japan hokkaido slimming pills[2]	Capsule		×
S1	A-Slim	Capsule	×	
S2	Best Shown coffee	Coffee bag	×	
S3	Dr Mao slimming capsules	Capsule	×	
S4	Fruit & plant slimming capsule	Capsule	×	
S5	Fruta bio	Capsule	×	
S6	Herbal Flos lonicerae	Capsule	×	
S7	Lingzhi cleansed slim tea	Tea bag	×	
S8	Lipo 8 burn Slim	Capsule	×	
S9	Shoufsy	Capsule	×	
S10	Slim-vie	Capsule	×	
S11	SLIMXTREM	Capsule	×	
S12	Via Slim	Capsule	×	
PS1	7 Days herbal Slim	Capsule	×	×
PS2	Fruta planta (grape)	Capsule	×	×
PS3	Fruta planta (grape)	Capsule	×	×
PS4	Pineapple plus	Capsule	×	×
PS5	Seven super color Slim & Health	Capsule	×	×
PS6	Seven super color Slim & Health	Capsule	×	×
PS7	Seven super color Slim & Health	Capsule	×	×
PS8	Shape	Capsule	×	×

PS9	Super Slim	Capsule	×	×
PS10	Li Da	Capsule	×	×
PS11	St Nirvana	Capsule	×	×
T1	7 days herbal slim	Capsule		
T2	Extra slim	Capsule		x
T3	Slim Xtreme Gold	Capsule		
T4	Lida daidaihua	Capsule		
T5	Apidessum	Capsule		
T6	Super slim	Capsule		×
T7	Figure-up	Capsule		×
T8	SlimBio Capsules	Capsule		
T9	Nuozitai	Capsule	×	
T10	Daidaihua	Capsule		
T11	NuoMeiRin	Capsule		
T12	Lipo 9	Capsule	×	
T13	St nirvana herbal slimming	Capsule	×	
X1	Acaï boost	Capsule	Raspberry ketone	
X2	Perfect Slim	Capsule	Fluoxetine	

[1] N, non-adulterated (natural) group; P, phenolphthalein-adulterated group; S, sibutramine-adulterated group; PS, both sibutramine and phenolphthalein-adulterated group); T, test samples, i.e. newly purchased slimming dietary supplements; X, atypical samples.

[2] This dietary supplement was erroneously reported as containing sibutramine in the paper of our group (Hachem et al., Proton NMR for detection, identification and quantification of adulterants in 160 herbal food supplements marketed for weight loss. J. Pharm. Biomed. Anal. 2016, 124, 34-47). It actually contains phenolphthalein as the adulterant.
